# Oncology training and education initiatives in low and middle income countries: a scoping review

**DOI:** 10.3332/ecancer.2021.1296

**Published:** 2021-09-30

**Authors:** Safiya Karim, Zahra Sunderji, Matthew Jalink, Sahar Mohamed, Indranil Mallick, Susan Citonje Msadabwe-Chikuni, Nancy J Delgarno, Nazik Hammad, Scott Berry

**Affiliations:** 1 Department of Medical Oncology, University of Calgary, Tom Baker Cancer Centre, 1331 29 St NW, Calgary, AB T2N 4N2, Canada; 2 Department of Family Medicine, University of British Columbia, Vancouver, BC V6T 2A1, Canada; 3 Department of Oncology, Queen’s University, Kingston, ON K7L 5P9, Canada; 4 Department of Public Health Sciences, Queen’s University, Kingston, ON K7L 3N6, Canada; 5 Department of Radiation Oncology, Tata Medical Center, Kolkata 700 160, India; 6 Cancer Diseases Hospital, Lusaka 10101, Zambia; 7 Office of Professional Development and Educational Scholarship, Faculty of Health Sciences, Queen’s University, Kingston, ON K7L 0E9, Canada

**Keywords:** medical education, low and middle-income countries, e-health, oncology, scoping review

## Abstract

**Background:**

The global cancer burden falls disproportionately on low and middle-income countries (LMICs). One significant barrier to adequate cancer control in these countries is the lack of an adequately trained oncology workforce. Oncology education and training initiatives are a critical component of building the workforce. We performed a scoping review of published training and education initiatives for health professionals in LMICs to understand the strategies used to train the global oncology workforce.

**Methods:**

We searched Ovid MEDLINE and Embase from database inception (1947) to 4 March 2020. Articles were eligible if they described an oncology medical education initiative (with a clear intervention and outcome) within an LMIC. Articles were classified based on the target population, the level of medical education, degree of collaboration with another institution and if there was an e-learning component to the intervention.

**Findings:**

Of the 806 articles screened, 25 met criteria and were eligible for analysis. The majority of initiatives were targeted towards physicians and focused on continuing medical education. Almost all the initiatives were done in partnership with a collaborating organisation from a high-income country. Only one article described the impact of the initiative on patient outcomes. Less than half of the initiatives involved e-learning.

**Conclusions:**

There is a paucity of oncology training and education initiatives in LMICs published in English. Initiatives for non-physicians, efforts to foster collaboration within and between LMICs, knowledge sharing initiatives and studies that measure the impact of these initiatives on developing an effective workforce are highly recommended.

In 2018, it was estimated that 18.1 million new cancer cases and 9.6 million cancer deaths occurred worldwide [1]. By 2040, the World Health Organization (WHO) estimates that global cancer cases will reach 29.5 million and there will be 16.5 million cancer related deaths [2]. Although high income countries (HICs) have higher cancer incidence rates, cancer mortality rates are significantly higher in low and middle-income countries (LMICs) (defined by the World Bank as countries with a gross national income of $12,535 or less in 2019 [3]) and continue to rise [4]. The proportion of cancer deaths in LMICs is expected to increase to 75% by 2030 [2, 5].

One of the major barriers to cancer control in LMICs is training an adequate oncology workforce [6]. On average, LMICs have 1.3 physicians and 2.5 nurses per 1,000 people compared with 3.1 physicians and 10.9 nurses per 1,000 people in HICs [7, 8]. As a result, case volumes and clinical workload are significantly higher for oncology providers in LMICs compared with HICs [9]. There are significant shortages in the oncology workforce, including specialists with skills in pathology, radiation and medical oncology [6]. Furthermore, the absence of sufficient oncology training programmes and lack of focus on cancer care during medical school provide significant challenges towards training and retaining cancer care professionals [10]. Given the rising cancer burden and limited workforce, it is essential to develop, promote and support local oncology education and training initiatives in LMICs.

Some traditional approaches to building the oncology workforce in LMICs such as travelling to HICs to acquire necessary skills may have unintended harmful consequences to LMIC health care systems [10]. The ‘brain drain’, when trainees do not return to their native country to practice medicine, has been recognised by the WHO as a major problem that propagates imbalances in the global health workforce [11]. To combat this, several global oncology initiatives have focused on building local capacity to provide high-quality cancer care [10]. One such mechanism involves building partnerships between institutions facilitating bi-directional learning and knowledge transfer, and enabling local retention and capacity building [10]. Furthermore, e-learning is increasingly being used to supplement learning in LMICs. Given the absence of a systematic examination of oncology education and training initiatives in LMICs, this scoping review was performed.

## Methods

Scoping reviews facilitate the examination of a broad topic of interest with the purpose of identifying gaps in the evidence, clarifying concepts and synthesising what is known about a topic [12]. We selected a scoping review methodology due to the paucity of empirical literature on this subject as well as to provide an overview of available information regarding oncology education and training in LMICs. This review aims to provide a description of the depth and breadth of the educational initiatives within this field [13]. We applied a rigorous approach to this scoping review through a five-step approach to ensure reliability: (1) identify the research question, (2) identify relevant studies, (3) select studies, (4) chart the data and (5) collate, summarise and report the results [13, 14].

### Identify the research question

Our scoping review was centred on the following research question: What has been published on oncology education and training to healthcare professionals in LMICs between the years 1947 and 2020?

### Identify relevant studies

A qualified medical librarian assisted in the development and execution of the search strategy in two major electronic databases: Ovid MEDLINE and Embase up to 4 March 2020 (date of the search). We felt that these two databases would capture the majority of the published literature in this field and that including additional databases would not yield a significant increase in the number of results. The search string is shown in Supplementary Appendix A. The reference lists of relevant review papers and articles found during the search of the databases were also scanned for additional eligible articles.

### Select studies

A two-stage standardised screening process was used to evaluate the eligibility of the articles identified in the search using Covidence systematic review software (Covidence Systematic Review Software 2019). Two independent reviewers (MJ and SM) screened the title and abstracts of the captured articles using the pre-defined eligibility criteria. The same reviewers screened the full-text articles that passed stage 1 review. If there was uncertainty as to whether an article should be included, a third author was asked to provide input (SK).

We applied pre-determined eligibility criteria to the selection process. Eligible articles described a specific oncology medical education initiative targeted towards individuals residing in a LMIC. The initiative must have described one or more specific interventions as well as an outcome of that intervention. Oncology education was broadly defined to include any teaching within the following disciplines: surgical oncology, medical oncology, radiation oncology, haematologic oncology, gynaecological oncology and/or topics: research skills, pathology, diagnosis, treatment, supportive care or palliative care. We excluded any article that did not meet the above criteria as well as review articles, letters, commentaries, book chapters, abstracts and unpublished manuscripts.

### Chart the data

Data were extracted using a pre-developed data extraction tool which included the terms: author, year, geographical area, host county(ies), host site/institution, collaborations, partner country, partner institution, location of training, project title, target population, level of education, use of e-learning, oncology discipline, programme duration (i.e. short (up to 1 month), medium (>1 month to 1 year), long-term (>1 year)), educational outcome(s) and funding. For the purposes of this study, e-learning was defined as learning facilitated by electronic technologies and involving access to a curricula outside of a traditional classroom, often in the form of interactive multimedia, audiovisual clips and virtual models [15].

### Collate, summarise and report the results

A descriptive numerical summary of the characteristics of the studies was performed. Tables were created to show the overall number of studies included, the target study population, level of medical education, publication year, use of e-learning, oncology discipline, programme duration, educational outcome(s) and funding. As per scoping review methodology, an assessment of the quality of the included studies was not performed [15].

We also classified the impact of the studies’ based on the 4-level model by Kirkpatrick that measures the outcomes of a particular education study or training programme [16]. These levels include: (1) *reaction,* (2) *learning,* (3) *behaviour* and (4) *results*.

## Results

**Figure 1.**
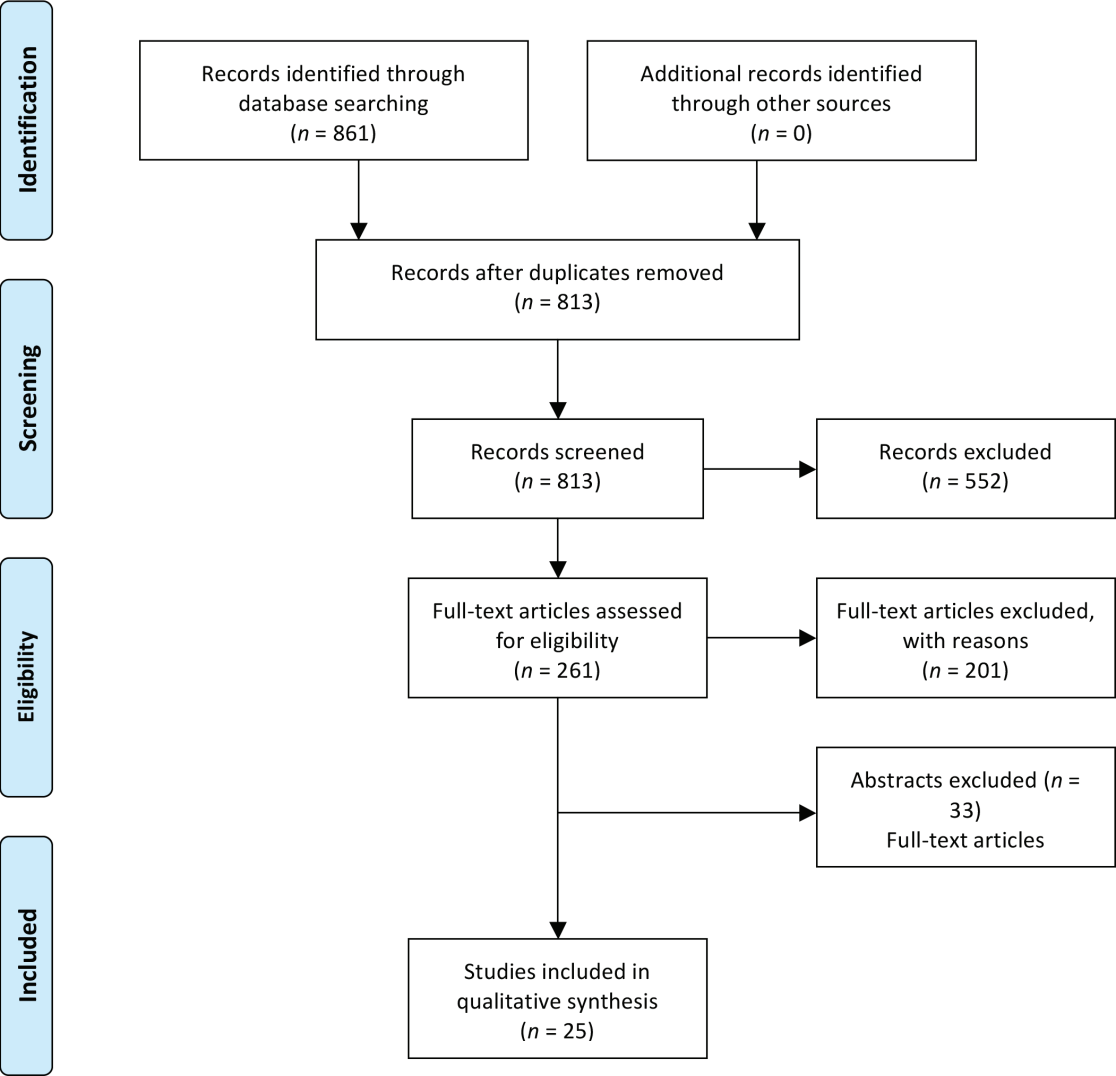
PRISMA (Preferred Reporting Items for Systematic Reviews and Meta-Analyses) chart.

After removal of duplicates, the initial search yielded 806 articles. Of these, 544 did not describe a specific medical education intervention. The remaining 261 full text articles were assessed for eligibility, and 201 did not meet the eligibility criteria. Sixty articles met our inclusion criteria, of which 33 were only in abstract form. Two articles were not available in full-text format. Twenty-five full-text articles were included in this scoping review (Figure 1). Of the 25 articles included, nine were quantitative studies [17–25], eleven were qualitative studies [27–36] and five were mixed method studies [37–41]. An overview of the selected articles is available in Table 1. Full details are available in Supplementary Tables 1 and 2.

### Target population and location

Fifteen of the twenty-five articles described an education initiative for physicians [17, 20, 23–25, 27, 29, 30, 33, 35–40]. Four articles described an initiative for nurses [19, 21, 28, 31], and one article was aimed at pharmacists [26]. Five articles described initiatives for more than one member of the health care team: three initiatives were for physicians and nurses [18, 22, 32], one was for physicians, nurses and pharmacists [41] and one was for physicians, nurses and other non-health professionals [34].

**Figure 2.**
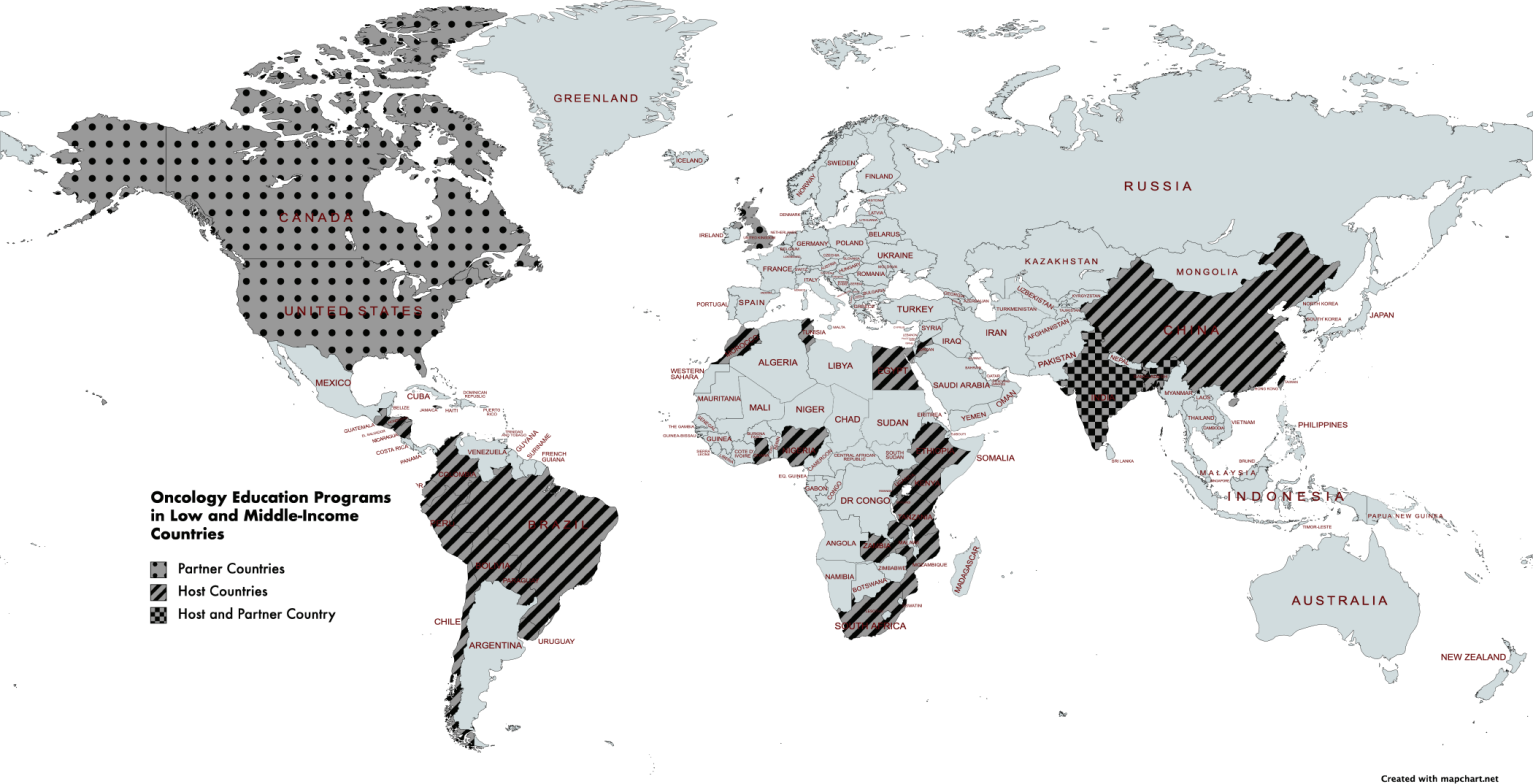
Low-and-middle income countries with educational initiatives highlighted in the 25 studies.

Thirteen initiatives were conducted in the Middle East/Africa [17, 19, 20, 25, 26, 29, 32, 34, 35, 38–41], six in the Americas [18, 23, 24, 27, 28, 31] and five in Asia/Pacific [21, 30, 33, 36, 37]. Project ECHO, a tele-mentoring programme for cervical cancer prevention was conducted in all three of these regions (Figure 2) [22].

### Level, topic and duration of education initiative

Continuing medical education (CME) initiatives (8 of 15) were the most common level of medical education for physicians, followed by graduate medical education (GME) initiatives (3 of 15), and a combination of CME/GME initiatives (2 of 15). There were only two initiatives for medical students (undergraduate medical education (UME)) [23, 33]. All four initiatives targeting nurses [19, 21, 28, 31] and the one initiative for pharmacists [26] were CME.

The initiatives focused on a range of oncology disciplines and topics. There were four initiatives in gynaecologic oncology (all in cervical cancer) [19, 22, 34, 39], four in medical/haematologic oncology [21, 29, 30, 41], four in paediatric oncology [18, 28, 31, 35], two in radiation oncology [17, 37], one in pathology [24] and one in surgical oncology [36]. There was one initiative that focused on both surgical oncology and pathology [20]. In addition, there was one intervention related to supportive care [26] and one on conducting population health research [27]. There were six initiatives that focused on multiple topics within oncology [23, 25, 32, 33, 38, 40]. There were nine short-term [20, 23, 25, 26, 31–34, 39], six medium-term [19, 24, 27, 28, 36, 40] and eight long-term initiatives [18, 21, 22, 29, 30, 35, 37, 41]. Two articles did not specify the length of their initiative [17, 38].

### Use of e-learning

Eleven articles described a component of e-learning in their educational intervention. Six studies included the use of telehealth technology [18, 22, 24, 26, 35, 37], one used smartphone-based training [19], two used online teaching modules [17, 39] and one used simulations [28].

### Collaborations

Almost all (24 of 25) educational initiatives were implemented with a collaborating organisation. The collaborating organisations were most often located in HICs, including fourteen from the United States [17, 19–22, 24–27, 29–32, 40], three from Canada [18, 35, 39], one from both the United States and Canada [28], two from the United Kingdom [38, 41] and one from Hong Kong [36]. Two articles described collaborations with a variety of international agencies, including the WHO [33, 34], and the International Agency for Research on Cancer (IARC) [34]. The article by Agrawal *et al* [37] was the only study to describe an in-country collaboration between two different institutions in India. Twelve articles noted that the collaboration between institutions was highly rewarding and beneficial in fostering bi-directional learning [18, 20, 21, 24, 30, 32, 34–36, 38, 40, 41].

The majority of the educational initiatives took place solely within the host LMIC (i.e. in-country training). Five initiatives had both in-country and out-of-country components to their training programmes, which involved travel to the collaborating organisation [24, 27, 29, 36, 41]. Only one initiative described an intervention that was solely located out-of-country, where participants spent the entire 3–6 weeks training at the collaborating organisation [40].

### Capacity building

Seven studies focused on building local capacity through their medical education initiative. Five initiatives implemented a ‘train the trainer’ model where learners who had completed the educational intervention previously would then serve as the instructor (‘trainer’) for the next cohort of learners [27, 28, 34, 36, 38]. This was done to build capacity and self-sustainability within the LMIC. Barron *et al* [21] described the future of their educational programme to include a focus on preceptorship development so that nurses would become skilled teachers thereby helping to ensure that the training programme becomes self-sustaining. Fish *et al* [40] describe the potential to build local capacity as a result of their educational intervention by laying the foundation for continued international research collaborations and connections.

### Impact of educational initiative

Based on the Kirkpatrick programme evaluation training model’s 4-levels of impact, 10 articles exclusively measured reaction outcomes [18, 22, 23, 26, 27, 29, 32, 33, 36, 37]. These were assessed primarily though surveys using Likert-scale, multiple choice and/or open-ended questions. Seven articles exclusively measured learning outcomes [17, 19, 20, 25, 30, 31, 34], most commonly through pre/post written tests. Two articles exclusively measured behaviour outcomes [35, 41]. For example, Lewis and Tibenderana [41] showed that after a series of training sessions in the safe handling, preparation and administration of chemotherapy, there was an increase in the rate of documented chemotherapy prescriptions which were correctly administered to patients compared to the pre-training levels. Three articles reported both reaction and learning outcomes [28, 38, 40], two articles learning and behaviour outcomes [24, 39] and one article reported learning and results outcomes [21]. The result outcome in this article pertained to the success of a bone marrow transplant programme in Bangladesh in the months following training of nurses.

### Challenges

Several challenges were identified in implementing the educational initiative. Language barriers [17, 27, 34] and the lack of protected time for the learners to participate in the initiative were often identified [27, 28, 31]. Four articles also noted encountering technical problems [19, 22, 26, 37] (i.e. Internet connectivity, videoconferencing equipment dysfunction) and four articles described infrastructure limitations (i.e. limited medical supplies, lack of accurate medical records) within the LMIC [19, 20, 22, 38]. The logistics and expenses of international travel was identified as a challenge in three articles [27, 29, 40]. Cultural differences and political/workforce changes were also highlighted in one article [38]. Eleven articles did not disclose their challenges in the implementation of the initiative [20–24, 28, 29, 31, 38, 40, 41].

### Funding

The majority of the initiatives were partially or fully funded by external grants and/or by a charitable foundation located in the partner country [17, 18, 22, 24, 27, 31, 34, 36]. One initiative was partially funded by the collaborating country’s institution [19] and one initiative was partially funded by the collaborating country’s government [41] while another was funded by a grant through the host country’s government [37]. Other sources of funding included research institutes [22, 23], a pharmaceutical company [22] (partial funding) and a fellowship award [32]. Ten articles did not disclose the source of funding for their initiative [20, 21, 25, 26, 28, 30, 33, 35, 38, 39].

## Discussion

Medical education and training initiatives in oncology are a critical element in developing an effective workforce to manage rising cancer incidence and mortality rates in LMICs. In this scoping review, we identified 25 articles that described an oncology medical education initiative. The majority of these initiatives were aimed at physicians and CME. Almost all the initiatives were in partnership with a collaborating organisation from a HIC. In addition, based on Kirkpatrick’s 4-level model, the majority of initiatives evaluated only reaction outcomes. Finally, less than half of the initiatives involved a component of e-learning.

**Figure 3.**
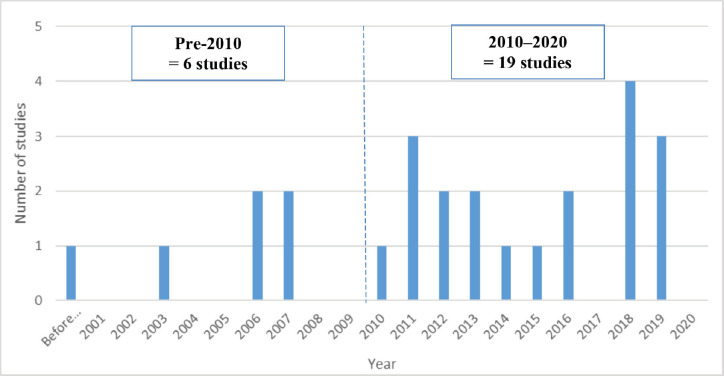
Number of published oncology medical education initiatives per year.

With only 25 articles identified, there is a clear paucity of published literature on educational initiatives in the field of oncology. This may be due to an absence of studies investigating this topic, or these initiatives are not being widely published in the peer-reviewed medical literature. For example, in LMICs, the emphasis may be on implementing an educational programme, and due to time, financial and/or language constraints, publication of such work, at least in full-text format, may not be possible. Over time, we found a gradual increase in the number of published manuscripts, with only six articles published before 2010 compared to 19 between 2010 and 2020 (Figure 3). This increase is encouraging as publication allows others to learn important lessons for design and implementation of medical education initiatives in oncology in LMICs. Where possible, efforts to assist educators in overcoming obstacles to conducting and publishing studies related to their initiatives could be considered. On the other hand, less onerous methods of disseminating important findings should also be encouraged.

The majority of the initiatives included in our scoping review were aimed towards physicians in LMICs. While the training of physicians is important, well-trained and skilled allied health professionals, including nurses and pharmacists, are also critical for the effective delivery of cancer care [42]. Education and training of other non-physician providers is integral to the workforce crisis in LMICs, and is necessary in order to build a strong cancer-care delivery infrastructure. Furthermore, inter-professional education and collaboration has been proposed by the WHO as a promising solution to ensure the appropriate supply, mix and distribution of the global health workforce [43]. Only five studies in our scoping review targeted multiple members of the healthcare team [18, 22, 32, 34, 41], showing that further focus on inter-professional education initiatives is warranted.

Our study also found that the majority of initiatives were focused on CME. This type of medical education is important for practicing health care providers to maintain competence and learn about new and developing areas within their field. However, if foundational education in oncology is not being provided in the earlier stages of health professions education, individuals may not have adequate exposure to the field in order to choose it as a speciality or profession. The lack of focus on oncology in medical school curricula in LMICs may have been due to an increased focus on infectious diseases, which historically has been the major concern in these countries [6]. However, with the growing burden of cancer in LMICs, it is also important to include cancer as a core discipline in undergraduate health professions education.

Over the past few decades, there has been increased interest from institutions from HICs to form partnerships with those from LMICs [46]. However, there is cause for concern that in many cases these partnerships may be driven by the funding and interests of the HIC partner, and that the needs of the LMIC institution are not fully considered [44]. Other concerns with these ‘North-South’ partnerships have also been raised, including the low degree of sustainability and a focus on short-term goals instead in long-term capacity building [45]. While several of the initiatives identified in our review had long-term partnerships and there was a focus on capacity building, almost all of these partnerships were with organisations from HICs. In recent years, ‘South-South’ collaborations (i.e. from within the same LMIC or between different LMICs) have gained popularity in the delivery of health professionals education due to several advantages, including better alignment with local needs, ability to engage senior leaders within the institutions and ensuring long-term sustainability [46].

The vast majority of the studies in our scoping review used either reaction or learning outcomes as their method of evaluation. These represent the two lowest levels of impact in Kirkpatrick’s framework [16]. These evaluation methods are the most easily measured and implemented, but also are the least effective over time. Conversely, behaviour and result outcomes are more difficult to implement, take longer to measure and are often more costly. However, the goal of any medical educational initiative should be to improve patient outcomes, which are evaluated in level four of the Kirkpatrick model. As oncology medical education initiatives become more prevalent, and as collaborations develop into longer-term relationships, it will be important that all four levels of Kirkpatrick’s model are evaluated to improve the impact of these initiatives.

LMICs require effective and affordable medical education strategies to address the limited and poorly trained workforce. In a recent systematic review of e-learning for medical education in LMICs from 2007 to 2017, Barteit *et al* [47] included 52 articles and concluded that e-learning in LMICs has not met its expected potential. They proposed that some reasons for the limited success of e-learning implementation may be due to financial resources and that educational organisations may not be giving e-learning a strong mandate as an educational method. Our findings, where only 11 of the 25 studies used a component of e-learning as part of their educational initiative, support the conclusion that e-learning may still be underutilised as an educational method in LMICs. Similarly, in a scoping review of e-learning for primary healthcare, Reeves *et al* [48] found that of 23 studies published on this topic, only two were from LMICs. E-learning initiatives have the potential to offer highly effective, low-cost and high-quality education, especially in resource limited settings but will likely require a combined effort and commitment from a variety of national and international stakeholders in order to meet its full potential [47].

Our study should be interpreted in the context of certain limitations. First, we employed a scoping review methodology that does not assess the rigour or quality of the included studies. Second, we chose to include only two databases (Ovid MEDLINE and Embase) and excluded programmes in the gray literature (i.e. abstracts), where many educational initiatives may have been highlighted. Finally, despite efforts to optimise the search terms in this scoping review, we may have missed certain medical education initiatives that were indexed under different terms.

## Conclusion

In conclusion, oncology training and education initiatives for health care professionals in LMICs are an essential component in building the workforce to address the growing cancer burden and to ensure that the workforce is well-trained to provide excellent care to cancer patients. This scoping review identifies a broad range of initiatives that have been published and the strategies used to implement them. In the coming years, we recommend that educators share valuable lessons learned related to the creation and execution of their programmes, including, where possible, performing and publishing studies of their work in order to optimise educational strategies. This may be achieved by establishing an online registry of oncology educational initiatives occurring across various LMICs and/or organising regional/national focused meetings that aim to share knowledge on best practices for delivering educational programmes. Furthermore, the design and delivery of oncology education for non-physician health professionals and between various health professionals should be prioritised to ensure optimal collaboration and teamwork within the global oncology workforce. Finally, in addition to long-term partnerships with HIC organisations, we encourage organisations within LMICs to foster collaborative relationships within their country or from other LMICs to better address local needs, contexts and ensure long-term sustainability of their educational initiatives.

## Funding

This manuscript was supported by the University of Calgary Global Oncology Programme.

## Conflicts of interest statement

The authors have no conflicts of interest.

## Figures and Tables

**Table 1.** Overview of the 25 manuscripts included in the scoping review.

First author	Year published	Low/middle income country	Collaborating country/organisation	Target population	Level of medical education	Duration	E-learning (Y/N)
Abugideiri [17]	2019	Ethiopia	USA	Physicians	GME	Not specified	Y – on-line teaching module
Adler [18]	2015	Barbados/Jamaica/St. Lucia	Canada	Physicians and nurses	CME	18 months	Y – telehealth
Agrawal [37]	2011	India	India	Physicians	GME	2 years	Y – telehealth
Alfaar [26]	2012	Egypt	USA	Pharmacists	CME	3 days	Y – telehealth
Arnold [27]	2014	Guatemala	USA	Physicians	CME	1 year	N
Asgary [19]	2016	Ghana	USA	Nurses	CME	3.5 months	Y – smartphone application
Awuah [20]	2011	Ghana	USA	Physicians	CME	1 week	N
Barron [21]	2018	Bangladesh	USA	Nurses	CME	3 years	N
Butonzi [29]	2019	Rwanda	USA	Physicians	CME	2 years	N
Carbone [30]	1991	Taiwan	USA	Physicians	GME	2 years	N
Day [31]	2012	Guatemala	USA	Nurses	CME	10 days	N
De Camargo [23]	2018	Brazil	N/A	Physicians	UME	1 day	N
Eastin [38]	2018	Kenya/Tanzania/Uganda	United Kingdom	Physicians	GME/CME	Not specified	N
Elit [39]	2010	Kenya	Canada	Physicians	CME	2 weeks	Y – on-line teaching module
Fish [40]	2019	South Africa/Tanzania/Rwanda	USA	Physicians	GME/CME	3–6 weeks	N
Kapoor [33]	2006	India	WHO	Physicians	UME	1 week	N
Lewis [41]	2019	Uganda	United Kingdom	Physicians, nurses, pharmacists	CME	18 months	N
Lopez [22]	2017	Multiple countries^a^	USA	Physicians and nurses	CME	2 years	Y – telehealth
Miller [34]	2007	Nigeria	Multiple organisations^b^	Physicians, nurses, non-health professionals	CME	10 days	N
Nwogu [32]	2014	Nigeria	USA	Physicians and nurses	CME	3 days	N
Qaddoumi [35]	2007	Jordan	Canada	Physicians	CME	17 months	Y – telehealth
Santiago [24]	2012	Brazil	USA	Physicians	CME	6 months	Y – telehealth
Shah [25]	2006	Egypt/Tunisia	USA	Physicians	CME	30 minutes	N
Tam [36]	2013	China	Hong Kong	Physicians	CME	1 year	N
Wilimas [28]	2003	Morocco/El Salvador/Guatemala	USA/Canada	Nurses	CME	3 months	Y – simulations

a Includes Brazil, Uruguay, Chile, Paraguay, Bolivia, Peru, Ecuador, Colombia, Nicaragua Honduras, India, South Africa, Mozambique, Nigeria, Ghana, and Zambia

b Includes American College of Clinical Pharmacy, WHO, IARC, Program for Appropriate Technology in Health, Engender Health, John Hopkins Program for International Education in Gynecology and Obstetrics (JHPIEGO) and Pan American Health Organization

**Supplementary Table 1.** Detailed summary of the 25 articles included in the scoping review.

Abugideiri [17]	2019	A Prospective International Pilot Study Evaluating the Efficacy of a Self-Guided Contouring Teaching Module with Integrated Feedback for Transitioning from 2D to 3D Treatment Planning	Middle East/Africa	Ethiopia	Black Lion Hospital/Addis Ababa University	USA	Winship Cancer Institute of Emory University	In LMIC
Adler [18]	2019	Bridging the Distance in the Caribbean: Telemedicine as a means to build capacity for care in paediatric cancer and blood disorders	Americas	Barbados, Jamaica, St. Lucia	University of the West Indies, Bustamante Hospital for Children	Canada	The Hospital for Sick Children	In LMIC
Agrawal [37]	2011	Training the trainees in radiation oncology with telemedicine as a tool in a developing country: A two-year audit	Asia Pacific	India	Chhatrapati Shahuji Maharaj Medical University	India	Sanjay Ghandi Post Graduate Institute of Medical Sciences	In LMIC
Alfaar [26]	2012	International telepharmacy education: another venue to improve cancer care in the developing world	Middle East/Africa	Egypt	Children’s Cancer Hospital, Cairo	USA	St. Jude Children’s Research Hospital	In LMIC
Arnold [27]	2014	A training programme to build cancer research capacity in low- and middle-income countries: findings from Guatemala	Americas	Guatemala	Instituto de Cancerologia (INCAN)	USA	School of Medicine of Washington University in St. Louis	In LMIC and HIC
Asgary [19]	2016	mHealth to train community health nurses in visual inspection with acetic acid for cervical cancer screening in Ghana	Middle East/Africa	Ghana	Korle Bu Teaching Hospital	USA	New York University	In LMIC
Awuah [20]	2011	Implementation of a percutaneous core needle biopsy training program: Results from the University of Michigan-Komfo Anokye Teaching Hospital breast cancer research partnership	Middle East/Africa	Ghana	Komfo Anokye Teaching Hospital (KATH), Kumasi	USA	University of Michigan	In LMIC
Barron [21]	2018	Building specialized nursing practice capacity in Bangladesh: An educational program to prepare nurses to care for oncology and bone marrow transplant patients in Dhaka, Bangladesh	Asia Pacific	Bangladesh	Dhaka Medical College Hospital and Massachusetts General Hospital	USA	Massachusetts General Hospital	In LMIC
Butonzi [29]	2019	Global Oncology Fellowship Electives: The Impact on Cancer Care and International Collaborations	Middle East/Africa	Rwanda	Butaro Hospital Cancer Center of Excellence	USA	University of Pennsylvania Center for Global Cancer Medicine, Geisel School of Medicine	In LMIC
Carbone [30]	1991	Developing a Postgraduate Medical Oncology Training Program in Taipei, Taiwan, Republic of China	Asia Pacific	Taiwan	Taipei	USA	University of Wisconsin	In LMIC
Day [31]	2012	A sustainable model for pediatric oncology nursing education in low-income countries	Americas	Guatemala	Unidad Nacional de Oncologia Pediatrica	USA	St. Jude Children’s Research Hospital	In LMIC
De Camargo [23]	2018	A sustainable model for pediatric oncology nursing education in low-income countries	Americas	Brazil	Multiple Medical Schools in Brazil	No partner country	N/A	In LMIC
Eastin [38]	2018	Addressing the burden of cancer in East Africa through cascaded training	Middle East/Africa	Kenya, Tanzania, Uganda	N/A (individual physicians)	UK	Royal College of Physicians, London, UK	In LMIC
Elit [39]	2010	Teaching Cervical Cancer Surgery in Low-or Middle-Resource Countries	Middle East/Africa	Kenya	Moi University	Canada	Gynaecologic Oncologists of Canada	In LMIC
Fish [40]	2019	POETIC (Program for Enhanced Training in Cancer): An Initial Experience of Supporting Capacity Building for Oncology Training in Sub-Saharan Africa	Middle East/Africa	South Africa, Tanzania, Rwanda	University of Cape Town, Ocean Road Cancer Centre	USA	Massachusetts General Hospital, Beth Israel Deaconess Medical Centre	In HIC
Kapoor [33]	2006	The UICC/WHO-CCCE Cancer Education Project: An Indian Experience	Asia Pacific	India	Gandhi Medical College, Bhopal	International	WHO Collaborating Center for Cancer Education, University Medical Center, The Netherlands	In LMIC
Lewis [41]	2019	Improving the safety of chemotherapy treatment for cancer patients in Uganda	Middle East/Africa	Uganda	Mbarara University of Science and Technology	UK	University of Bristol	In LMIC and HIC
Lopez [22]	2017	Project Echo: A Telementoring Program for Cervical Cancer Prevention and Treatment in Low-Resource Settings	Americas, Middle East/Africa, Asia Pacific	Multiple countries including Brazil, Uruguay, Chile, Paraguay, Bolivia, Peru, Ecuador, Colombia, Nicaragua Honduras, India, South Africa, Mozambique, Nigeria, Ghana, Zambia	Multiple organisations	USA	University of New Mexico, University of Texas MD Anderson Cancer Center	In LMIC
Miller [34]	2007	Knowledge dissemination and evaluation in a cervical cancer screening implementation program in Nigeria	Middle East/Africa	Nigeria	University of Ibadan	International Organisations	Multiple organisations including ACCP, WHO, IARC, PATH, Engender Health, JHPIEGO, PAHO	In LMIC
Nwogu [32]	2014	Promoting Cancer Control Training in Resource Limited Environments: Lagos, Nigeria	Middle East/Africa	Nigeria	Lagos State University Teaching Hospital	USA	Roswell Park Cancer Institute	In LMIC
Qaddoumi [35]	2007	Impact of Telemedicine on Pediatric Neuro-Oncology in a Developing Country: The Jordanian-Canadian Experience	Middle East/Africa	Jordan	King Hussein Cancer Center	Canada	The Hospital for Sick Children	In LMIC
Santiago [24]	2012	Improving the histopathologic diagnosis of pediatric malignancies in a low-resource setting by combining focused training and telepathology strategies	Americas	Brazil	Instituto Materno Infantil de Pernambuco	USA	The Mayo Clinic and St. Jude Children’s Research Hospital	In LMIC and HIC
Shah [25]	2006	Knowledge gained after a brief CME module on breast cancer diagnosis	Middle East/Africa	Egypt/Tunisia	Individual primary care physicians from Egypt and Tunisia	USA	University of Michigan School of Public Health and Cancer Center, and the University of Texas M.D Anderson Cancer Centre	In LMIC
Tam [36]	2013	Internationalization: the Hong Kong-China experience as a model for collaborative education in Asia	Asia Pacific	China	University of Medical Sciences Beijing, Shanghai Second Medical University	Hong Kong	University of Hong Kong	In LMIC and HIC
Wilimas [28]	2003	Training subspecialty Nurses in Developing Countries: Methods, Outcome, and Cost	Americas	Morocco, El Salvador, Guatemala	Morocco – Not Reported (NR), El Salvador – Benjamin Bloom Hospital Guatemala – NR	USA and Canada	St. Jude Children’s Research Institute, Children’s Hospital of Eastern Ontario, The Hospital for Sick Children	In LMIC

ACCP, American College of Clinical Pharmacy; WHO, World Health Organization; IARC, International Agency for Research on Cancer; PATH, Program for Appropriate Technology in Health; PAHO, Pan American Health Organization

**Supplementary Table 2.** Detailed description of the 25 manuscripts included in the scoping review (Part 2).

Abugideiri [17]	Physicians	GME	Y	Radiation Oncology	Not specified	Learning	Host country institution and National Cancer Institute Grant
Adler [18]	Physicians and nurses	CME	Y	Paediatric oncology	18 months	Reaction	Sick Kids Foundation
Agrawal [37]	Physicians	GME	Y	Radiation oncology	2 years	Reaction	Grant by Department of Science and Technology, India
Alfaar [26]	Pharmacists	CME	Y	Supportive care	3 days	Reaction	NR
Arnold [27]	Physicians	CME	N	Population Health Research	1 year	Reaction	National Institutes of Health and National Cancer Institute
Asgary [19]	Nurses	CME	Y	Gynaecologic oncology (cervical cancer)	3.5 months	Learning	New York University Institute of Global Health, Global Health Challenge Fund
Awuah [20]	Physicians	CME	N	Surgical oncology and pathology	1 week	Learning	NR
Barron [21]	Nurses	CME	Y	Medical and haematologic oncology	3 years	Learning and results	NR
Butonzi [29]	Physicians	CME	N	Hematologic oncology	2 years	Reaction	Personal time (i.e. vacation time)
Carbone [30]	Physicians	GME	N	Medical Oncology	2 years	Learning	NR
Day [31]	Physicians and nurses	CME	N	Paediatric oncology	10 days	Learning	National Cancer Institute, American Lebanese Syrian Associated Charities
De Camargo [23]	Physicians	UME	N	Multiple disciplines	1 day	Reaction	CEPHO (research centre affiliated with a medical school) and self-funded through registration cost
Eastin [38]	Physicians	GME/CME	N	Multiple disciplines	Not specified	Reaction and Learning	NR
Elit [39]	Physicians	CME	Y	Gynaecologic oncology (cervical cancer)	2 weeks	Learning and Behaviour	NR
Fish [40]	Physicians	GME/CME	N	Multiple disciplines	3–6 weeks	Reaction and Learning	Eddie Reed Philanthropic Fund
Kapoor [33]	Physicians	UME	N	Multiple disciplines	1 week	Reaction	NR
Lewis [41]	Physicians, nurses, pharmacists	CME	N	Medical oncology	18 months	Behaviour	UK Government Department for International Development
Lopez [22]	Physicians and nurses	CME	Y	Gynaecologic oncology (cervical cancer)	2 years	Reaction	National Institutes of Health, Cancer Prevention Research Institute of Texas, Cancer Prevention Pharmaceuticals
Miller [34]	Physicians, nurses and non-health professionals	CME	N	Gynaecologic oncology (cervical cancer)	10 days	Learning	National Cancer Institute, ExxonMobil Foundation, T. Boone Pickens Research Fund
Nwogu [32]	Physicians and nurses	CME	N	Multiple disciplines	3 days	Reaction	UICC International Cancer Technology Transfer Fellowship funded by ASCO
Qaddoumi [35]	Physicians	CME	Y	Paediatric oncology	17 months	Behaviour	NR
Santiago [24]	Physicians	CME	Y	Pathology	6 months	Learning and behaviour	National Cancer Institute, American Lebanese Syrian Associated Charities
Shah [25]	Physicians	CME	N	Multiple disciplines	30 minutes	Learning	NR
Tam [36]	Physicians	CME	N	Surgical oncology (paediatric)	1 year	Reaction	S.K Yee Medical Foundation
Wilimas [28]	Nurses	CME	Y	Paediatric oncology	3 months	Reaction and learning	NR

UME, Undergraduate medical education; GME, Graduate medical education; CME, Continuing medical education; NR, Not reported; CEPHO, Centro de Estudos e Pesquisa de Hematologia e Oncologia; UICC, Union for International Cancer Control; ASCO, American Society of Clinical Oncology

## Supplementary Appendix A. Search Strategy for Embase and Ovid

Database: Embase Classic+Embase <1947 to 04 March 2020> Search strategy:

1. developing country/ (95112)
2. (developing adj2 countr*).mp. (137447)
3. (low income adj2 countr*).mp. (12243)
4. (middle income adj2 countr*).mp. (22509)
5. (lmic or lmics or lami countr*).mp. (5522)
6. exp China/ (221918)
7. exp India/ (150498)
8. exp Indonesia/ (17899)
9. exp Brazil/ (113496)
10. exp Pakistan/ (27316)
11. Nigeria/ (38503)
12. Bangladesh/ (16005)
13. exp Mexico/ (48224)
14. Philippines/ (11907)
15. Ethiopia/ (16430)
16. Sri Lanka/ (8639)
17. Kenya/ (21526)
18. Tanzania/ (15257)
19. Ghana/ (11602)
20. Thailand/ (33928)
21. Viet Nam/ (16730)
22. Nepal/ (11528)
23. Senegal/ (7000)
24. Uganda/ (18201)
25. Rwanda/ (3575)
26. or/1-25 (879016)
27. exp socioeconomics/ (384867)
28. low income.mp. (44302)
29. middle income.mp. (25281)
30. 28 or 29 (63027)
31. 27 and 30 (41592)
32. gross national product/ (4289)
33. 30 and 32 (384)
34. 31 or 33 (41675)
35. 26 or 34 (899172)
36. medical student/ (69251)
37. exp physician/ (770756)
38. exp medicine/ (3516532)
39. resident/ (41748)
40. fellow*.mp. (41637)
41. clinical clerkship.mp. (658)
42. or/36-41 (4170864)
43. education/ (432964)
44. ((staff? or profession*) adj2 develop*).mp. (25018)
45. preceptorship.mp. (1174)
46. exp teaching/ (98612)
47. curriculum/ (90598)
48. exp medical education/ (331141)
49. videoconferencing/ (3653)
50. webcast/ (341)
51. e-learn*.mp. (4368)
52. exp telemedicine/ (38155)
53. or/43-52 (823448)
54. 35 and 42 and 53 (19929)
55. exp oncology/ or oncology.mp. (301657)
56. 54 and 55 (777)

Database: Ovid MEDLINE(R), Ovid MEDLINE(R) Daily and Epub Ahead of Print, In-Process & Other Non-Indexed Citations <1946 to Present> Search Strategy:

1. developing countries/ (73860)
2. (developing adj2 countr*).mp. (124238)
3. (low income adj2 countr*).mp. (6581)
4. (middle income adj2 countr*).mp. (17390)
5. (lmic or lmics or lami countr*).mp. (4439)
6. exp China/ (183472)
7. exp India/ (100850)
8. Indonesia/ (10128)
9. Brazil/ (90177)
10. Pakistan/ (17021)
11. Nigeria/ (27823)
12. Bangladesh/ (10604)
13. Mexico/ (37549)
14. Philippines/ (8182)
15. Ethiopia/ (12083)
16. Sri Lanka/ (5858)
17. Kenya/ (15502)
18. tanzania/ (11010)
19. ghana/ (7867)
20. thailand/ (25929)
21. vietnam/ (11941)
22. nepal/ (7872)
23. senegal/ (5630)
24. uganda/ (11691)
25. rwanda/ (2330)
26. or/1-25 (687275)
27. exp Socioeconomic Factors/ (441733)
28. low income.mp. (34224)
29. middle income.mp. (19404)
30. 28 or 29 (50443)
31. 27 and 30 (20396)
32. gross domestic product/ (800)
33. 30 and 32 (73)
34. 31 or 33 (20427)
35. 26 or 34 (700368)
36. Students, Medical/ (32911)
37. exp Physicians/ (137284)
38. exp Medicine/ (1104890)
39. “Internship and Residency”/ (48445)
40. fellow*.mp. (29263)
41. Clinical Clerkship/ (4981)
42. or/36-41 (1260910)
43. Education/ (20633)
44. ((staff? or profession*) adj2 develop*).mp. (22740)
45. preceptorship/ (5016)
46. exp Teaching/ (84291)
47. exp Curriculum/ (83607)
48. exp Education, Medical/ (162473)
49. exp Videoconferencing/ (1756)
50. webcast/ (865)
51. e-learn*.mp. (2568)
52. exp Telemedicine/ (27252)
53. or/43-52 (324612)
54. 35 and 42 and 53 (7390)
55. exp Medical Oncology/ or oncology.mp. (114202)
56. 54 and 55 (80)
